# The Countess of Dufferin's Fund

**Published:** 1888-03-03

**Authors:** 


### THE COUNTESS OF DUFFERIN'S FUND.

The first of the silver medals presented by H. E. the Viceroy to the National Association for Supplying Female Medical Aid to the Women of India has been claimed by the authorities of the Grant Medical College, Bombay, for presentation to Miss B. Bradley, the most successful student during the past year at the first examination in the certificated practitioners class.

Sir Walter DeSouza, whose liberality has enabled many female students to study at the Calcutta Medical College, has just placed a cheque for Rs. 2,400 in the hands of the Central Committee, being his final donation to the sum which, under the name of the DeSouza Trust, has conferred so much good upon scholars and sick in the Indian metropolis.

The Queen's Register is nearly finished, but the number of contributors to the Jubilee collection were so great that it will yet be some days before the book will be ready for despatch to her Majesty, as every entry has to be most carefully examined. A copy of the list is being made out for printing, and the names of all contributors, together with a copy of the presentation address, will be published with the annual report.

The capital engraving in a recent number of the *Graphic* of Dr. Barter's class of nurses at Nagpur, Central Provinces, shows that interest in the movement has extended beyond the limits of the Indian Empire. The reports from the branches are now being received by the Central Committee, and give a very satisfactory account of the progress of the association during the past twelve months. The provincial organisation is working well, and the local committees are settling down steadily to their work. The co-operation of the medical authorities and the various municipalities and district boards is very noticeable in the reports of the branches. The interest taken in the movement by the non-official members of the various local boards and committees is one of the most encouraging signs of public approval.

The third annual general meeting will be held in Calcutta early in February, and it is hoped that it may be attended by representatives of the various branches. There are not any changes to propose in the rules of the association, but it is very probable that it may be found necessary to incorporate the association in view of the increasing responsibility incurred in the distribution and custody of public money.

Viceroy's Camp, India.

### "HE GIVETH SLEEP."

Of all the thoughts of God that are
Borne inward into souls afar
Along the Psalmist's music deep,
Now tell me if that any is
For gift or grace surpassing this—
"He giveth his belovèd, sleep?"

What would we give to our beloved?
The hero's heart to be unmoved,
The poet's star-tuned harp to sweep,
The patriot's voice to teach and rouse,
The monarch's crown to light the brows?
He giveth his belovèd, sleep.

What do we give to our beloved?
A little faith all undisproved,
A little dust to over-weep,
And bitter memories to make
The whole earth blasted for our sake:
He giveth his belovèd, sleep.

O earth, so full of dreary noises!

O men—with wailing in your voices!

O delved gold, the wailers heap!

O strife, O curse, that o'er it fall!

God strikes a silence through you all,

And giveth his belovèd, sleep.

—*Elizabeth Barrett Browning.*

